# Atherosclerotic cardiovascular disease and mortality in a cohort of patients with rheumatoid arthritis: a prospective study investigating microRNAs as predictors of atherosclerosis and mortality

**DOI:** 10.3389/fimmu.2025.1667553

**Published:** 2025-10-08

**Authors:** Dídac Llop, Silvia Paredes, Daiana Ibarretxe, Roser Rosales, Lluís Masana, Josep Ribalta, Joan Carles Vallvé

**Affiliations:** ^1^ Unitat de Recerca en Lípids i Arteriosclerosi, Facultat de Medicina i Ciències de la Salut, Universitat Rovira i Virgili, Reus, Spain; ^2^ Institut d’Investigació Sanitària Pere Virgili, Reus, Spain; ^3^ Centro de Investigación Biomédica en Red de Diabetes y Enfermedades Metabólicas Asociadas (CIBERDEM), Instituto de Salud Carlos III (ISCIII), Madrid, Spain; ^4^ Rheumatology Section, Sant Joan University Hospital, Reus, Spain; ^5^ Vascular Medicine and Metabolism Unit, Sant Joan University Hospital, Reus, Spain

**Keywords:** rheumatoid arthritis, epigenetics, mortality, microRNAs, atherosclerotic cardiovascular disease

## Abstract

Rheumatoid arthritis (RA) is a chronic autoimmune disorder associated with an increased risk of atherosclerotic cardiovascular disease (ASCVD) that is not fully explained by traditional risk factors. This study investigated whether a novel microRNA (hsa-miR) panel could improve cardiovascular risk prediction and stratification in RA patients. In this 8-year prospective cohort study, 235 RA patients were enrolled, of whom 148 completed follow-up. We quantified six hsa-miRs (hsa-miR-24, -146, -Let7a, -425, -451, and -155-5p) using qPCR and evaluated their predictive value for two primary endpoints: ASCVD progression (new atherosclerotic plaques and/or non-fatal cardiovascular events) and all-cause mortality using partial least squares discriminant analysis (PLS-DA), linear mixed models, and multivariate regression. During follow-up, 58 patients (39%) experienced ASCVD progression, and 35 died (ASCVD accounting for 31% of deaths). PLS-DA models indicated that baseline hsa-miR levels predicted both ASCVD progression and mortality, explaining 43% and 42% of outcome variability, respectively. Longitudinal changes in five hsa-miRs (-24, -146, -let-7a, -425, and -155-5p) also predicted ASCVD progression. Age, hypertension, and disease duration modulated hsa-miR expression levels over time. This hsa-miR panel represents a promising tool for improving cardiovascular risk prediction in RA, potentially addressing critical gaps in current stratification approaches. Following validation, it could support implementation of personalized cardiovascular risk assessment in RA clinical practice.

## Graphical Abstract

MicroRNAs as predictors of atherosclerotic cardiovascular disease and mortality in rheumatoid arthritis.

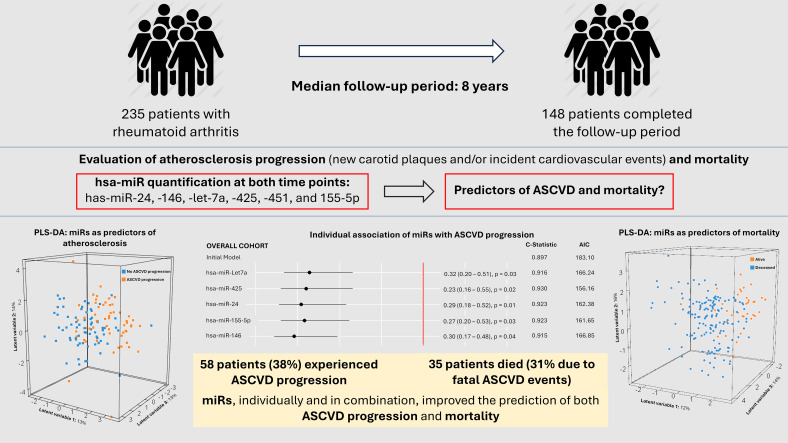

## 1 Introduction

Rheumatoid arthritis (RA) is a chronic autoimmune disease characterised by synovial inflammation, leading to cartilage and bone erosion, functional disability, and increased mortality (1). Systemic inflammation in RA also predisposes patients to extra-articular complications, including an increased risk of developing atherosclerotic cardiovascular disease (ASCVD), the leading cause of death in this population. Individuals with RA face a 50% higher risk of ASCVD than the general population, primarily due to the instability and rupture of atherosclerotic plaques (2–4). Although traditional risk factors such as smoking, obesity, and hypertension are more prevalent in RA, they do not fully explain the heightened ASCVD risk, underscoring the need to identify novel biomarkers to enhance risk prediction and understand underlying mechanisms.

MicroRNAs (hsa-miRs) have emerged as key regulators in the pathophysiology of various diseases, including ASCVD (5–7). These small, non-coding RNAs negatively regulate gene expression post-transcriptionally. To prevent rapid degradation in extracellular fluids, hsa-miRs are transported within microvesicles, exosomes, or lipoproteins, which enhance their stability and bioavailability (8). Several studies have identified specific hsa-miRs as potential biomarkers for acute myocardial infarction and coronary artery disease in the general population, potentially due to their roles in endothelial dysfunction and inflammatory responses (9–11). In a previous study, our group quantified 754 circulating hsa-miRs and identified a panel of 10 with similar expression patterns in patients with RA and in non-RA individuals with acute myocardial infarction, while both groups displayed distinct profiles compared with healthy controls (12), suggesting a potential role of these microRNAs in mediating ASCVD risk in RA. From this initial discovery, we subsequently focused on hsa-miR-24, -146a, -let-7a, -425, and -451, which have since been linked to systemic inflammation and subclinical atherosclerosis in RA (13–15). In addition, hsa-miR-155 has emerged as a key regulator of inflammation and immunity (16, 17). This miR functions as a master regulator of the immune response and inflammation and has been implicated in the pathogenesis of various diseases, including RA and cardiovascular disease, demonstrating inflammatory effects in plasma and foam cell formation and potential as a therapeutic target (18–21). However, the potential of these microRNAs as predictive biomarkers for ASCVD progression and mortality in longitudinal studies remains largely unexplored.

This prospective long-term study investigated ASCVD progression and overall mortality in a cohort of patients with RA over an 8-year period. The expression levels of a panel of hsa-miRs (hsa-miR-24, -146, -Let7a, -425, -451, and -155) as potential predictors of ASCVD progression and mortality in RA was assessed, with the aim of improving risk stratification and clinical management and providing insight into the interplay between hsa-miRs, inflammation, and atherosclerosis. In addition, we studied the biological variables most strongly associated with longitudinal changes in the expression of these selected hsa-miRs.

## 2 Materials and methods

### 2.1 Study design and participants

This was a prospective cohort study including 235 patients with RA, as previously described (13, 22, 23), recruited consecutively from outpatient clinics at Sant Joan University Hospital (Reus, Spain) between November 2011 and January 2015. Eligible participants were aged 18–80 years and had a confirmed diagnosis of RA based on the 1987 American College of Rheumatology (ACR) criteria, complemented by the 2010 ACR/EULAR classification to improve diagnostic specificity. Exclusion criteria included age <18 or >80 years, and the presence of acute intercurrent conditions such as neoplasia, chronic kidney disease, active infections, and other autoimmune diseases or significant comorbidities, including psoriatic arthritis, systemic lupus erythematosus, chronic liver disease, inflammatory bowel disease, or multiple sclerosis, among others. All participants provided blood samples and clinical data at baseline. Follow-up assessments were conducted between December 2020 and January 2022. During follow-up, patients who were reclassified with a different diagnosis, died, or were lost to follow-up were excluded from the longitudinal analysis. The final cohort included 148 patients with complete data at both time points and a median follow-up of 8 years. The study was approved by the Clinical Research Ethics Committee of our hospital (CEIm: 11-04-28/4proj5 for baseline and CEIm: 222/2020 for follow-up) and conducted in accordance with our institution’s guidelines and the Declaration of Helsinki.

### 2.2 Laboratory measurements

Data on classical cardiovascular risk factors (smoking, hypertension, type 2 diabetes mellitus (T2DM), and dyslipidaemia), history of ASCVD events, and medication use were collected. Clinical measurements included body mass index (BMI), systolic, and diastolic blood pressures. Disability was assessed using the Health Assessment Questionnaire (HAQ) index. Disease activity was measured with the disease activity score (DAS28), which was derived from the erythrocyte sedimentation rate (ESR) and the number of tender and swollen joints. DAS28-ESR variable was categorised into remission (<2.6), low activity (2.6-3.2), moderate activity (3.2-5), and high activity (>5). Rheumatoid factor (RF) positivity was defined as RF>20 UI/L, and anti-citrullinated protein antibody (ACPA) positivity as ACPA > 3 U/mL (BioPlex 2200 Anti-CCP, Bio-Rad Laboratories).

Blood samples were collected after at least 12 hours of fasting, using EDTA as an anticoagulant. Plasma was separated from the whole blood by centrifugation at 3000 rpm for 10 minutes and stored at -80°C for further analysis. Analytical assessments, performed using enzymatic and standard methods, included measurements of RF (Roche, Germany), ACPA (BioRad, USA), and inflammatory markers such as ESR (Diesse DiagnosticaSenese S.pA., Italy), CRP (Roche, Germany), and fibrinogen (Instrumentation Laboratory SpA, USA).

### 2.3 Study endpoints

The study endpoints were: (i) ASCVD progression, defined as the development of new carotid atherosclerotic plaques and/or the occurrence of non-fatal cardiovascular events (including coronary artery disease, stroke, or peripheral artery disease) during the follow-up period. Patients who developed either a new plaque and/or a non-fatal ASCVD event were classified as ASCVD progressors (code = 1), whereas those without new plaques or events were classified as non-progressors (code = 0). This composite endpoint was designed to capture in the same endpoint the different stages of atherosclerosis, reflecting its progressive nature (24). Incident ASCVD events were documented by the physician during follow-up visits, whereas carotid plaque presence was assessed using a MyLab 60 X-Vision sonographer (Esaote SpA, Genoa, Italy). A plaque was defined as a focal structure protruding into the arterial lumen by at least 0.5 mm or 50% of the surrounding intima-media thickness value, or as having a thickness greater than 1.5 mm (25); (ii) all-cause mortality, defined as death from any cause occurring during the follow-up period. To ensure robustness of the results, endpoints were also evaluated individually: carotid plaque progression and incident cardiovascular events.

### 2.4 Plasma microRNA expression

A panel of microRNAs, including hsa-miR-24, -146, -Let7a, -425, -451 and -155, were analysed in the baseline and followed-up samples. Prior to RNA extraction, haemolysis was assessed in 200 µl plasma aliquots using spectrophotometric analysis at λ=414 nm, corresponding to the oxyhaemoglobin absorption peak. Samples with haemolysis were excluded. RNA containing the small RNA fraction was extracted from 200 µL of frozen plasma using the commercial miRCURY RNA Isolation Kit (Exiqon), following the manufacturer’s instructions. Prior to extraction, 1 µL of a synthetic RNA mixture (UniSp2, UniSp4, and UniSp5; Exiqon) was spiked into plasma to monitor extraction efficiency. In addition, 1.25 µL of MS2 RNA carrier (Roche) was added to improve RNA recovery. The final RNA was eluted in 50 µL of nuclease-free water. Reverse transcription (RT) was performed using 2 µL of RNA in a 10 µL reaction with the miRCURY LNA Universal RT microRNA PCR and Universal cDNA Synthesis Kit II (Exiqon, Denmark). RT conditions were: 60 min at 42°C, 5 min at 95°C, and cooling at 4°C. RT efficiency was controlled by adding 0.5 µL of cel-miR-39-3p and UniSp6 (Exiqon). The resulting cDNA was diluted 1:40 before quantification by qPCR. Candidate microRNAs were quantified using the miRCURY LNA Universal RT microRNA PCR system, ExiLENT SYBR Green Master Mix Kit (Exiqon, Denmark), and specific commercial LNA™ PCR primer sets (UniRT). Amplification was performed on a 7900HT Fast Real-Time PCR System (Applied Biosystems) under the following conditions: 10 min at 95°C, followed by 40 cycles of 10 s at 95°C and 1 min at 60°C. A melting curve analysis was performed to verify the specificity of amplification (13). Hsa-miR-16-5p was selected as the reference for normalization, as it showed optimal stability after evaluation with RefFinder (26) in our previous studies (13, 14). Relative expression levels were calculated using the ΔCt method, where ΔCt = Ct (candidate hsa-miR) – Ct (hsa-miR-16-5p). The ΔCt method was selected to allow normalization to a stable endogenous control, consistent with established approaches in circulating hsa-miR studies (27, 28). A higher ΔCt value indicated a lower expression level of the candidate hsa-miR. Cycle threshold (Ct) values were obtained using SDS v2.3 software (Applied Biosystems, USA).

### 2.5 Statistical analysis

We conducted four main types of analyses:

1. Baseline plasma expression levels of the selected panel of hsa-miRs were jointly evaluated as predictors of the study endpoints (all-cause mortality and ASCVD progression) using partial least squares discriminant analysis (PLS-DA). PLS-DA relates the predictor matrix (X; independent variables) to the response variable (Y; ASCVD progression or mortality) by maximising group discrimination and the covariance between X and Y, enabling dimensionality reduction and visual interpretation. Latent variables (LVs), which are linear combinations of the predictors, are generated to optimise this relationship, providing insights into the contribution of individual variables to classification. We used the sparse variant of PLS-DA (sPLS-DA) to identify the most predictive variables per LV while ensuring model parsimony. In addition to the selected hsa-miRs, the sPLS-DA model included clinical covariates such as age, sex, BMI, disease duration, hypertension status, T2DM, dyslipidaemia, RA treatments, DAS28-ESR, and lipid-lowering therapies. Model performance was assessed using 5-fold cross-validation to estimate the area under the ROC curve (AUC) for each latent variable, and a bootstrap procedure (N = 1,000) was used to calculate error rates as an additional measure of classification accuracy. The study was designed with sufficient statistical power to detect meaningful differences in AUC for predicting mortality and ASCVD progression, requiring 151 and 78 individuals, respectively, based on expected event rates and conventional statistical thresholds (α = 0.05, power = 80%).
2. We then examined whether the baseline levels of each hsa-miR, analysed individually and independently, could predict the study endpoints. For this, we fitted multivariate logistic regression models adjusted for relevant confounders, including age, sex, BMI, disease duration, hypertension status, T2DM, dyslipidaemia, RA treatments and lipid-lowering therapies. Model performance was assessed using the C-statistic (AUC) to evaluate discriminative ability and the Akaike Information Criterion (AIC) to compare model fit, with lower AIC values indicating better performance. Sample size adequacy for these models was estimated using Cohen’s formula, based on the effect size derived from the proportion of variance explained by the predictors (α = 0.05; 80% power). This analysis indicated that a sample size of 50 participants was sufficient to detect significant associations between individual microRNAs and ASCVD progression.
3. We also investigated whether changes in hsa-miR levels over the follow-up period were predictive of the study endpoints using repeated measures analyses with generalized linear mixed-effects models for participants with data available at both time points. In these models, ASCVD progression was the dependent variable, while hsa-miR expression at both time points and relevant confounders were included as independent variables. To reduce model complexity and avoid overfitting, a confounder selection process was applied beforehand. Candidate variables included age, sex, BMI, disease duration, dyslipidaemia, T2DM, hypertension, RA treatments (including Janus kinase (JAK) inhibitors), and lipid-lowering therapies. A full model including all candidate confounders was first fitted to identify non-influential variables. Confounders with p-values >0.10 were excluded, while those with p-values ≤0.10 were retained for the final predictive model. Sample size estimation for the mixed-effects logistic regression model was performed using a simplified approximation for binary outcomes with repeated measures, as described by Diggle et al. (29). Assuming a within-subject correlation of 0.4, two repeated observations per patient, an α = 0.05, and 80% power, the minimum required sample size to detect an OR of 0.50 for hsa-miR expression and ASCVD progression, based on previous findings (14), was 106 subjects,
4. Finally, repeated measures analyses using linear mixed-effects models were conducted to identify the biological variables most strongly affecting hsa-miR expression over the follow-up period. Here, microRNA expression levels at both time points were the dependent variables, and independent variables included age, sex, BMI, disease duration, hypertension, dyslipidaemia, and T2DM. Model performance was assessed using the C-statistic and AIC.

For descriptive analyses, differences between baseline and follow-up variables were tested using the paired t-test for normally distributed data or the Wilcoxon signed-rank test for non-normal data. The McNemar test was used for dichotomous variables. Differences between men and women at baseline and follow-up were evaluated using the Student’s t test, the Mann–Whitney U test, or the chi–square (χ²) test, as appropriate. P-values <0.05 were considered statistically significant. All analyses were performed using R software, version 4.2.0.

## 3 Results

### 3.1 General characteristics at baseline and at follow-up

The baseline characteristics of the 235 patients initially included, as well as those of the 148 patients who completed follow-up, are summarised in **Table 1**. The median age at baseline was 57 years (IQR 49–67), with 64% of the cohort being women. At follow-up (median duration: 8 years), the median age was 63 years (IQR 56–69), and 68% were women. The prevalence of dyslipidaemia increased significantly over time (p = 0.005), while the proportions of patients with hypertension, T2DM, and current smoking remained stable. Triglyceride levels showed a slight increase, and LDL-C levels a modest decrease, although neither reached statistical significance (p=0.06 and p=0.08, respectively). Markers of disease activity and inflammation improved significantly: DAS28-ESR (p<0.001), ESR (p=0.01), and CRP (p=0.003) all decreased. The proportion of patients in remission increased (p=0.005), while those with moderate and high disease activity decreased (p=0.02 and p<0.001, respectively). Regarding treatment, the use of biologics (including JAK inhibitors) increased significantly (p<0.001), whereas the use of csDMARDs, NSAIDs, and corticosteroids decreased (p<0.05 for all). There was also a trend toward increased use of lipid-lowering therapies (p=0.06). Sex-stratified analyses (**Supplementary Tables 1**, **2**) showed that women had higher disease activity at baseline, which decreased at follow-up to levels similar to men. The prevalence of atherosclerotic plaques and cardiovascular events remained higher in men at both time points.

**Table 1:** General characteristics of the patients with RA included at baseline and at follow-up.

	Basal (n = 235)	Follow-up (n = 148)	p
Clinical and demographic			
Age (years, IQR)	57 (49 – 67)	63 (56 – 69)	< 0.001
Sex, female (n, %)	150, 64%	101, 68%	0.44
Body mass index (kg/m2, IQR)	27.1 (23.6 – 30.8)	26.5 (23.3 – 30.8)	0.40
Hypertension (n, %)	138, 59%	95, 64%	0.34
T2DM (n, %)	27, 11%	23, 16%	0.32
Dyslipidaemia (n, %)	96, 41%	83, 56%	0.005
Current smoker (n, %)	61, 26%	28, 19%	0.14
Lipidic profile			
Total cholesterol (mg/dL, IQR)	203 (182.5 – 227.5)	199.5 (170.8 – 228)	0.20
LDL-C (mg/dL, IQR)	114 (99 – 135.5)	110 (90 – 132)	0.08
HDL-C (mg/dL, IQR)	65 (52 – 76)	61.50 (51 – 78)	0.68
TG (mg/dL, IQR)	94 (70 – 127.5)	98 (77 – 140)	0.06
RA disease features and treatments			
Disease duration (years, IQR)	6 (2 – 13)	14 (10 – 20)	< 0.001
DAS28-ESR (median, IQR)	3.42 (2.6 – 4.4)	2.74 (2.2 – 3.7)	< 0.001
- Remission (n, %)	59, 25%	58, 39%	0.005
- Low activity (n, %)	45, 19%	40, 27%	0.10
- Moderate activity (n, %)	106, 45%	48, 32%	0.02
- High activity (n, %)	25, 11%	2, 1%	<0.001
DAS28-CRP (median, IQR)	2.12 (1.3 – 3.0)	1.95 (1.5 – 2.7)	0.81
RF+ (%, n)	173, 74%	118, 80%	0.21
ACPA+ (%, n)	167, 71%	116, 78%	0.14
ESR (mm/h, IQR)	30 (18 – 50)	24.50 (13.75 – 42)	0.01
CRP (mg/dL, IQR)	0.40 (0.2 – 0.9)	0.20 (0.1 – 0.6)	<0.001
csDMARDs (n, %)	175, 74%	84, 57%	<0.001
Biological agents (n, %)	46, 20%	71, 48%	<0.001
- JAK inhibitors	0, 0%	11, 7%	<0.001
NSAIDs (n, %)	137, 58%	18, 12%	<0.001
Corticoids (n, %)	122, 52%	45, 30%	<0.001
Lipid-lowering therapies (n, %)	42, 18%	39, 26%	0.06
Atherosclerotic cardiovascular disease progression			
Carotid plaque presence (n, %)	92, 39%	74, 50%	0.047
CV events (n, %)	18, 8%	20, 14%	0.09
ASCVD progression (n, %)		57, 39%	0.03

Comparison of the general characteristics, disease features, treatments and cardiovascular progression of the cohort at baseline and after 8 years.

IQR, interquartile range; T2DM, type 2 diabetes mellitus; LDL-C, low-density lipoprotein cholesterol; HDL-C, high-density lipoprotein cholesterol; TG, triglycerides; DAS28, disease activity score; RF, rheumatoid factor; ACPA, anti-citrullinated peptide antibodies; ESR, erythrocyte sedimentation rate; CRP, C-reactive protein; csDMARDS, conventional synthetic disease modifying antirheumatic drugs; NSAIDs, non-steroidal anti-inflammatory drugs; JAK, Janus Kinase; CV, cardiovascular; ASCVD, atherosclerotic cardiovascular disease.

### 3.2 ASCVD progression

Over the follow-up period, ASCVD progression was observed in 39% (n= 58) of patients (those who developed either a new plaque and/or suffered a non-fatal cardiovascular event). Overall, there was an absolute increase of 11% in carotid plaque prevalence, rising from 39% at baseline to 50% at follow-up (p = 0.047). The proportion of patients with non-fatal ASCVD events increased from 8% to 14% over the follow-up period (p = 0.09).

### 3.3 Mortality and causes of loss to follow-up

Of the 235 patients initially included in the cohort, 16 were reclassified with a different diagnosis, 36 were lost to follow-up (due to relocation, severe frailty precluding hospital visits, or voluntary withdrawal), and 35 died during the study period. Causes of death included infections (n=12, 34%), cardiovascular disease (n=11, 31%), neoplasms (n=7, 20%), and other causes (n=5, 14%).

**Supplementary Table 3** compares the baseline characteristics of patients who completed follow-up with those who died. Patients who died were older (p<0.001), more likely to be male (p=0.02), had a higher BMI (p=0.003), and showed a greater prevalence of hypertension (p=0.001) and T2DM (p=0.01). They also had higher levels of inflammatory biomarkers, including ESR (p=0.004) and CRP (p=0.01), although no significant differences were observed in lipid profiles or disease activity measures. Regarding ASCVD parameters, they also had a higher prevalence of carotid plaques (p<0.001) and cardiovascular events (p=0.01).

### 3.4 Prediction of ASCVD progression using baseline expression of the selected hsa-miR

**Figure 1:**
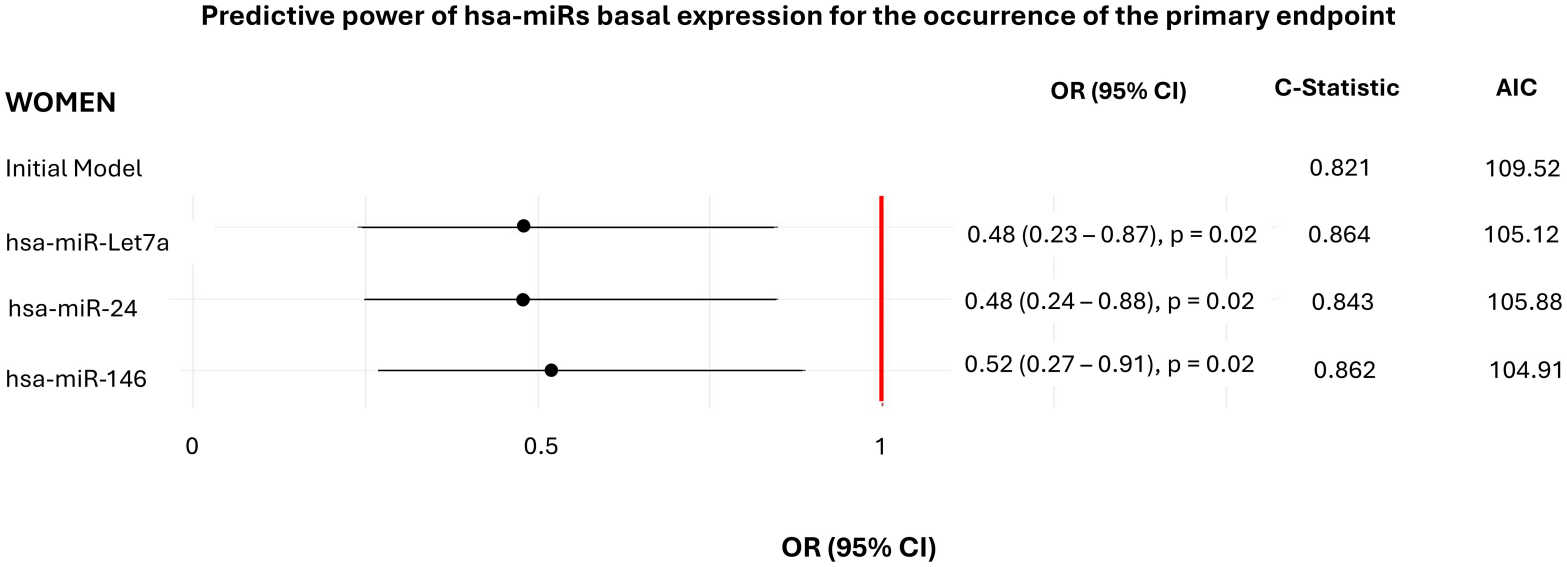
sPLS-DA model to predict ASCVD progression. Graphical representation of the Partial Least Square Discriminant Analysis (PLS-DA) model to predict ASCVD progression including all hsa-miRs and confounders (age, sex, BMI, disease duration, hypertension status, T2DM, dyslipidaemia, RA treatments, DAS28, and lipid-lowering therapies), along with the feature importance of each LV.

A sparse PLS-DA model was developed to assess the combined contribution of a panel of hsa-miRs for predicting ASCVD progression at follow-up in the entire cohort. The model selected the 10 mostrelevant variables for each LV. The resulting model (**Figure 1**) explained 40% of the total variability (LV-1 = 13%, LV-2 = 14%, LV-3 = 13%) and significantly discriminated between ASCVD progressors and non-progressors (p=0.001). Notably, hsa-miRs -24, -146, -Let7a, -425, -451 and 155-5p were key contributors to the model’s performance. The model showed fair discriminative accuracy, with mean AUCs of 0.76, 0.75, and 0.74 for LV-1 to LV-3, respectively, and moderate classification errors ranging from 0.33 to 0.31 across components. A bootstrap validation showed an overall mean error rate of 0.20 (95% CI: 0.13–0.29), supporting the robustness of the model’s predictive performance. When hsa-miRs were excluded in a sensitivity analysis (**Supplementary Figure 1**), the model’s predictive capacity declined, with total explained variability decreasing to 33% (LV1 = 19%, LV2 = 8%, LV3 = 6%) and a less clear separation between groups. Discriminative performance also slightly decreased, with mean AUCs of 0.76, 0.73, and 0.73 for LV1 to LV3, respectively, and marginally higher classification errors (ranging from 0.34 to 0.32 for all LV), supporting the added predictive value of circulating hsa-miRs for the classification of ASCVD progression in RA.

### 3.5 Prediction of ASCVD progression using individual baseline expression of hsa-miRs

**Figure 2:**
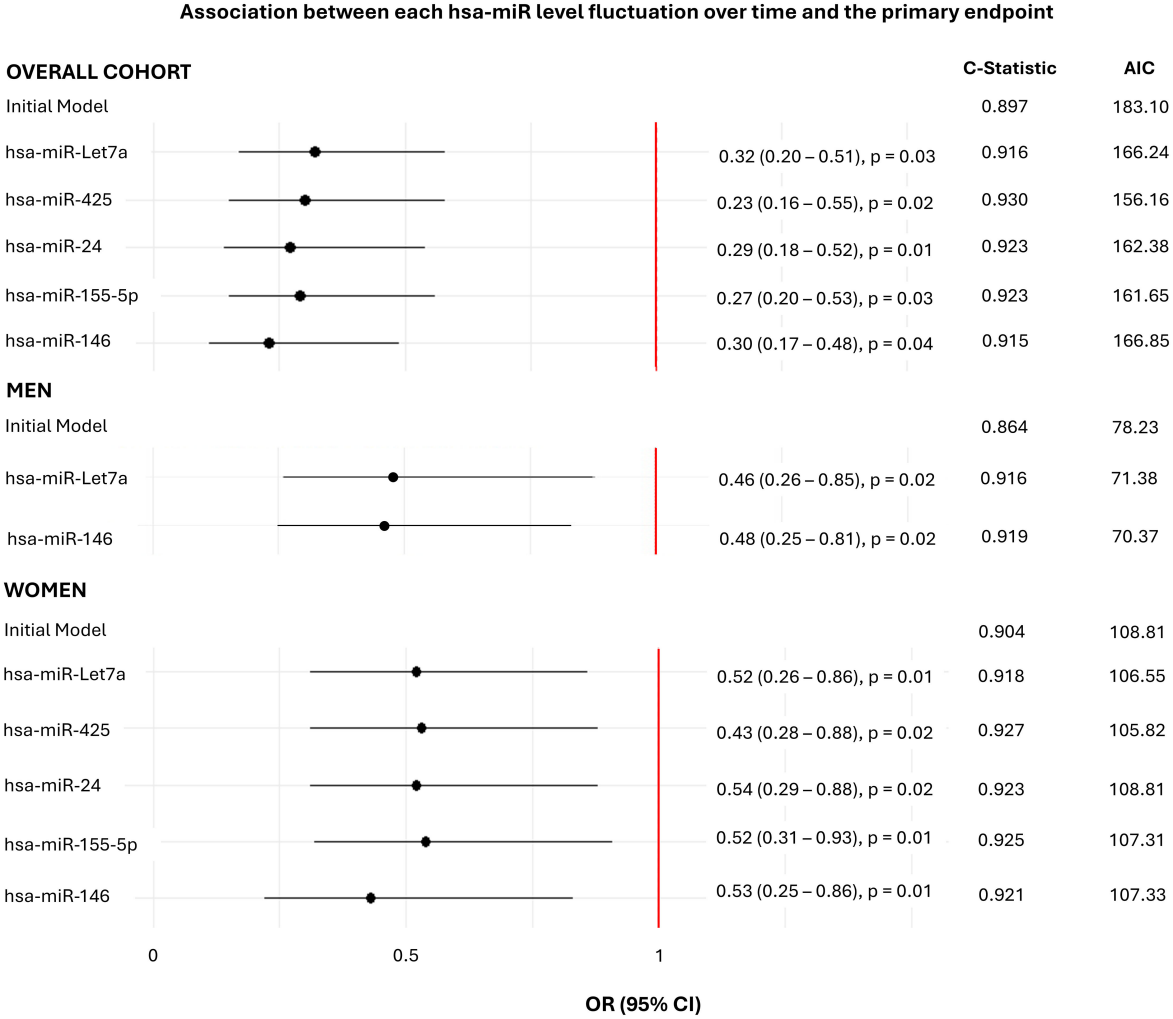
Associations between individual basal expression of each hsa-miR and ASCVD progression. Complete summaries of the logistic regressions evaluating the power of basal expression levels of the selected hsa-miRs, stratified by sex, on predicting ASCVD progression. Models are adjusted for age, sex, BMI, disease duration, hypertension, T2DM, dyslipidaemia, DAS28, RA treatment and lipid-lowering therapies. P < 0.05 are considered statistically significant. OR, odds ratio; CI, confidence interval; AIC, Akaike information criteria; hsa-miR, microRNA; ASCVD, atherosclerotic cardiovascular disease.

When baseline expression levels of each hsa-miR were evaluated individually, no significant associations with ASCVD progression were found in the overall cohort (**Figure 2**). However, in sex-stratified analyses lower baseline expression levels of hsa-miR-24 (OR = 0.48, p=0.02), -146 (OR = 0.52, p=0.02) and -Let7a (OR = 0.48, p=0.02) significantly predicted ASCVD progression in women. The inclusion of these hsa-miRs in the models enhanced predictive performance, as reflected by an increased C-statistic and a reduced AIC, thereby improving model accuracy (**Figure 2**). In contrast, no significant associations were observed in men. To ensure robust analyses, models were adjusted for age, sex, BMI, disease duration, hypertension status, T2DM, dyslipidaemia, DAS28, RA treatment and lipid-lowering therapies.

Baseline expression levels of hsa-miR-24, -146 -Let7a, and -425 were also individual predictors of carotid plaque progression in women, again improving model accuracy (**Supplementary Table 4**). Similarly, hsa-miR-146, -Let7a, and -155-5p were associated with incident cardiovascular events (**Supplementary Table 5**). Models consistently adjusted for the same set of clinical variables.

### 3.6 Prediction of ASCVD progression using longitudinal individual hsa-miR changes

**Figure 3:**
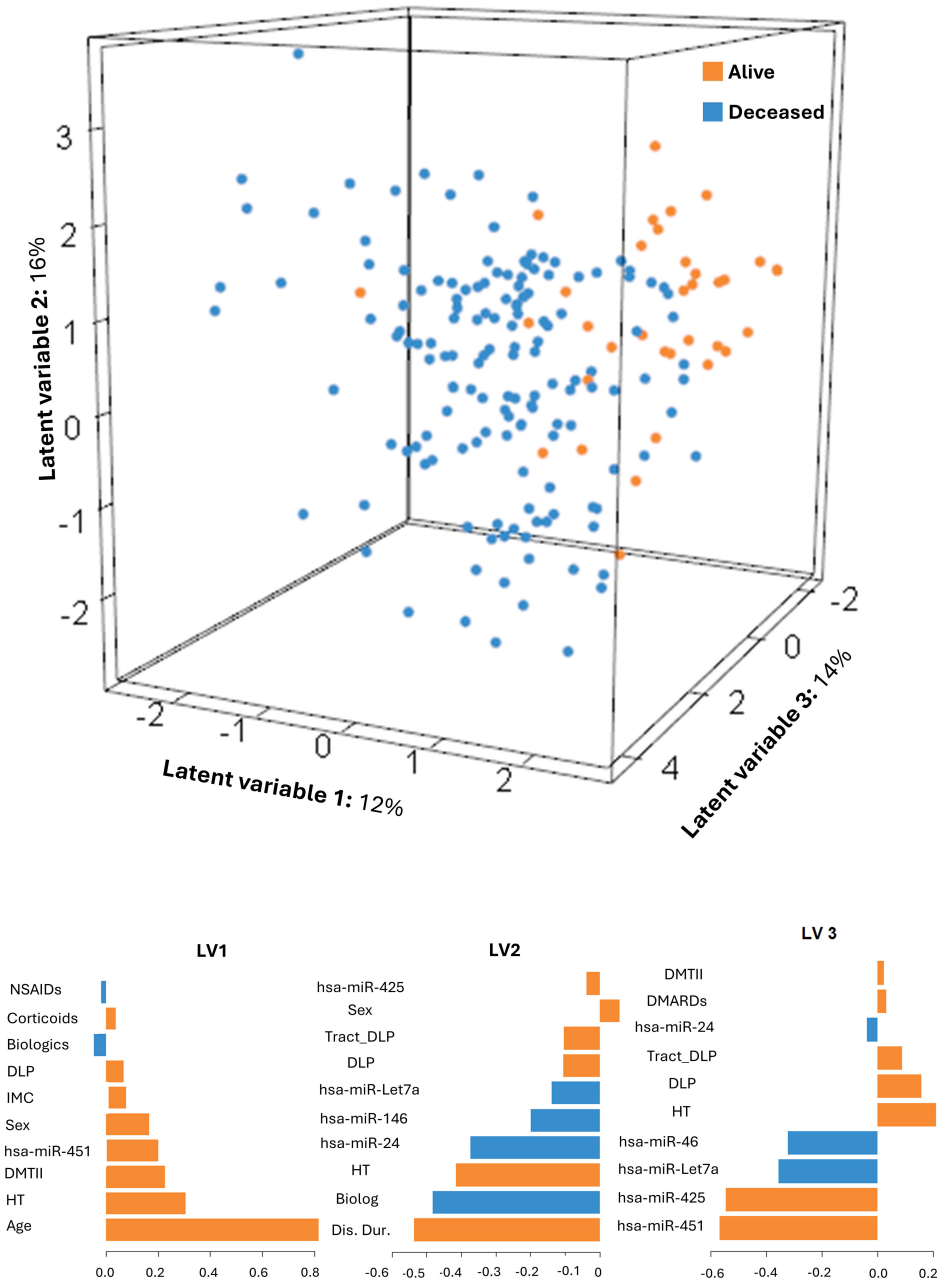
Association of the change of expression of each hsa-miR over time with ASCVD progression. Summaries of the generalized linear-mixed models assessing the association of each hsa-miR with ASCVD progression. The models were adjusted for age, sex, BMI, NSAID use, biologic use, lipid-lowering medication use, T2DM status, and DAS28. P < 0.05 are considered statistically significant. BMI, body mass index; T2DM, type 2 diabetes mellitus; ASCVD, atherosclerotic cardiovascular disease; AIC, Akaike Information Criteria.

Multivariate generalised linear mixed-effect models were fitted to assess whether changes in the expression of individual hsa-miRs over the follow-up period predicted ASCVD progression. The selection of the most influential confounders for these models are shown in **Supplementary Table 6**, and model summaries are presented in **Figure 3**. Including each hsa-miR in the initial model revealed that lower expression levels of hsa-miR-24 (OR = 0.29, p=0.01), hsa-miR-146 (OR = 0.30, p=0.04), hsa-miR-Let7a (OR = 0.32, p=0.03), hsa-miR-425 (OR = 0.23, p=0.02) and hsa-miR-155-5p (OR = 0.27, p=0.03) were individually associated with decreased odds of ASCVD progression. The role of these hsa-miRs was further demonstrated by an improvement in model performance upon their inclusion, as evidenced by an increased C-statistic and a reduced AIC, enhancing both classification and overall predictive capacity.

In sex-stratified analyses, all five hsa-miRs were predictive in women (hsa-miR-24: OR = 0.54, p=0.02; hsa-miR-146: OR = 0.53, p=0.01; hsa-miR-Let7a: OR = 0.52, p=0.01; hsa-miR-425: OR = 0.43, p=0.01); hsa-miR-155-5p: OR = 0.52, p=0.01) while only hsa-miR-146 (OR = 0.48, p=0.02) and hsa-miR-Let7a (OR = 0.46, p=0.01) were predictive in men.

The inclusion of these hsa-miRs in the generalized linear mixed models improved performance in both sexes, as reflected by an increased C-statistic and reduced AIC (**Figure 3**).

### 3.7 Prediction of mortality using baseline expression of the selected hsa-miRs

**Figure 4:**
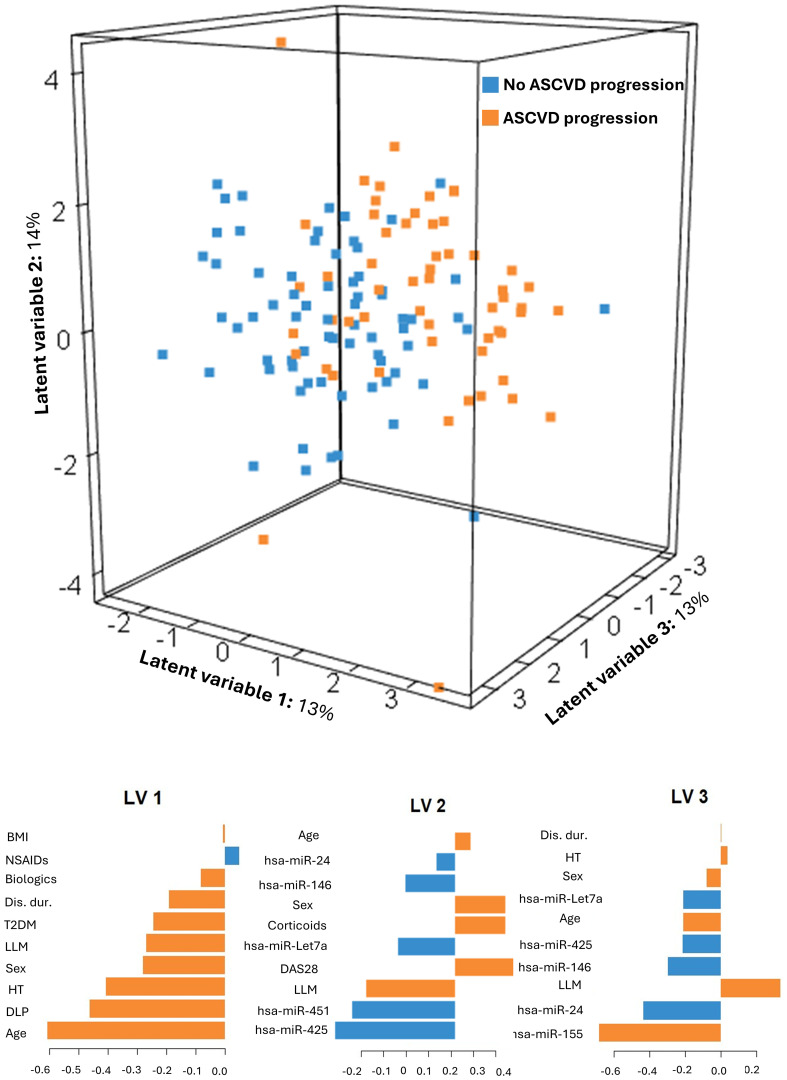
sPLS-DA model to predict the mortality. Graphical representation of the sPLS-DA model to predict mortality including all hsa-miRs and confounders (age, sex, BMI, disease duration, hypertension status, T2DM, dyslipidaemia, RA treatments, DAS28, and lipid-lowering therapies), along with the feature importance of each LV.

A sparse sPLS-DA model was developed to predict all-cause mortality using baseline levels of a selected hsa-miR panel (excluding hsa-miR-155-5p due to missing data) in combination with traditional CV risk factors. The resulting model (**Figure 4**) demonstrated strong discriminative capacity for classifying patients who died during follow-up, with AUCs of 0.85, 0.85, and 0.86 for LV1, LV2, and LV3, respectively (p=0.001). Overall, the model explained 42% of the total variability (LV1 = 12%, LV2 = 16%, LV3 = 14%). A bootstrap validation showed an overall mean error rate of 0.10 (95% CI: 0.06–0.15). Although classification errors were moderate (0.37, 0.30, and 0.28 for LV1 to LV3, respectively), the high AUC values indicated robust discrimination between patients who died and those who survived. Notably, LV2 and LV3 were enriched with the selected hsa-miRs, underscoring their contribution to the model’s predictive structure. When hsa-miRs were excluded in a sensitivity analysis (**Supplementary Figure 2**), the model’s predictive performance decreased, with total explained variability dropping to 37%. Discriminative accuracy was also reduced, reflected by slightly increased classification errors (0.39, 0.31, and 0.30 for LV1 to LV3, respectively) and marginally lower AUCs (0.83, 0.84, and 0.84 for LV1 to LV3, respectively), supporting the contribution of the selected circulating hsa-miRs as informative biomarkers that enhance the model’s capacity to predict mortality outcomes in RA patients.

Finally, baseline expression levels of individual hsa-miRs were not significantly associated with mortality risk in the overall cohort, nor were any significant associations observed in sex-stratified analyses.

### 3.8 Biological determinants of hsa-miR expression changes over the follow-up period

**Figure 5:**
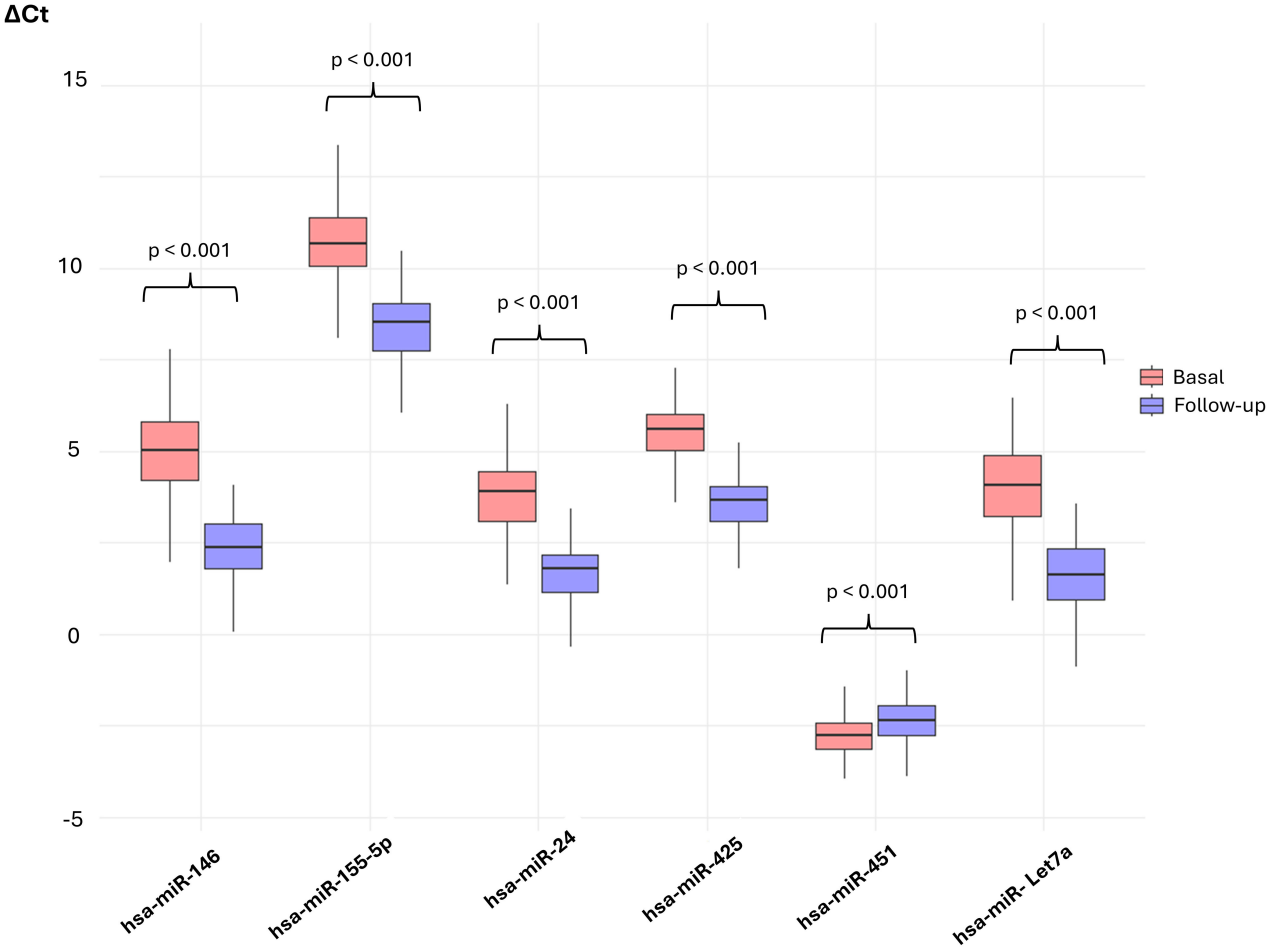
Expression levels of the selected hsa-miRs at baseline and at follow-up. Levels of expression of the selected hsa-miRs at baseline and at follow-up. Wilcoxon signed-rank test was used to compare the levels between both times. P < 0.05 are considered statistically significant.

Expression levels of hsa-miR-24, -146, -Let7a, -425, and -155-5p increased over the follow-up period (ΔCt decreased), while hsa-miR-451 expression decreased (ΔCt increased) (**Figure 5**).

Linear mixed-effects models identified age and dyslipidaemia as significant predictors of changes in the expression of hsa-miR-24 (β=-0.19, p=0.01; β=-0.32, p=0.01, respectively) and hsa-miR-146 (β=-0.18, p=0.001; β=-0.31, p=0.01, respectively). Disease duration and hypertension influenced hsa-miR-Let7a (β=-0.15, p=0.04; β=0.14, p=0.035, respectively) while BMI and disease duration affected hsa-miR-425 (β=0.17, p=0.008; β=-0.15, p=0.04, respectively). Sex was associated with changes in hsa-miR-451 (β=-0.26, p=0.04), and disease duration also influenced hsa-miR-155-5p (β=-0.16, p=0.04) (**Supplementary Table 7**).

## 4 Discussion

This 8-year prospective study investigated the progression of ASCVD and overall mortality in a cohort of 235 patients with RA, of whom 148 completed the follow-up. We further evaluated the predictive value of a panel of hsa-miR (hsa-miR-24, -146, -Let7a, -425, -451, and -155-5p) for these outcomes, both individually and in combination. In addition, we explored how longitudinal changes in hsa-miR expression levels were associated with ASCVD progression and identified biological factors that may underlie these fluctuations.

During the study, 16 patients were reclassified as having other autoimmune diseases, 36 were lost to follow-up due to relocation, fragility, or withdrawal, and 35 patients died. Diagnostic reclassification is not uncommon in RA, particularly in its early stages, as clinical features often overlap with other autoimmune conditions. Over time, disease progression typically reveals more distinct manifestations that help refine the diagnosis (30).

Infections were the leading cause of death (34%), followed by CVD (31%) and neoplasia (20%). The high incidence of infection-related deaths may, in part, reflect the overlap of the follow-up period with the COVID-19 pandemic, as RA is associated with worse COVID-19 outcomes (31, 32). Patients who died were older, more often male, and had a higher prevalence of cardiovascular comorbidities, such as hypertension and T2DM. They also exhibited elevated inflammatory markers (ESR and CRP), despite having similar DAS28-ESR scores compared to survivors. This suggests that low-grade subclinical inflammation, insufficiently captured by composite disease activity indices and potentially amplified by concomitant cardiometabolic conditions, may contribute to the increased mortality risk observed in RA (33, 34).

A key finding of our study is that adding the hsa-miR panel to sPLS-DA models that already included traditional cardiovascular risk factors and medication significantly improved the prediction of ASCVD progression (hsa-miRs -24, -146, -Let7a, -425, -451 and -155-5p) and overall mortality (hsa-miRs -24, -146, -Let7a, -425, and -451), increasing the explained variability by 7% and 5%, respectively, compared to models without these biomarkers. This underscores the added value of the hsa-miR panel for identifying patients at higher risk, consistent with previous studies demonstrating the superior predictive accuracy of hsa-miR panels over individual hsa-miRs (35, 36). Longitudinal analyses further revealed that lower expression levels of hsa-miR-24, -146, -Let7a, -425, and -155-5p were each independently associated with reduced odds of ASCVD progression, after adjustment for multiple confounders. These models showed good performance based on C-statistic and AIC. Sex-stratified analyses revealed that all tested hsa-miRs had significant predictive value for ASCVD progression in women, whereas only hsa-miR-146 and -Let7a were significant predictors in men. In women, lower baseline expression of hsa-miR-24, -146, and -Let7a was also significantly associated with reduced odds of ASCVD progression.

Circulating hsa-miRs are implicated in atherosclerosis in both the general population and in RA (5, 7). The specific selected hsa-miRs for this study are known regulators of key inflammatory and atherosclerotic pathways, including oxidative stress, endothelial dysfunction, angiogenesis and plaque stability (37–46). Among these, miR-24, miR-146, miR-155-5p, and miR-Let-7a have been described as protective against these processes, whereas miR-425 and miR-451 are often dysregulated in patients with established ASCVD. In RA, these hsa-miRs have previously been linked to markers of subclinical atherosclerosis, including increased carotid intima-media thickness and the presence of carotid plaques (13–15). However, to our knowledge, their predictive value for ASCVD progression and mortality in longitudinal studies has not been assessed.

From a clinical perspective, despite advances in disease management, likely due to the introduction of new targeted therapies such as JAK inhibitors and novel lipid-lowering agents, ASCVD progression, defined as the development new plaques and/or non-fatal cardiovascular events, significantly increased during the follow-up period. This underscores the urgent need for novel biomarkers to identify patients at high risk of atherosclerotic progression, enabling more personalized and intensive therapeutic strategies. Our results, pending further validation, highlight the potential of these hsa-miRs as biomarkers for predicting future ASCVD and worse clinical outcomes, as reflected by overall mortality. Notably, different associations were observed in sex-stratified analyses, suggesting differential roles of hsa-miRs in men and women. While the mechanisms remain incompletely understood, several factors may contribute to sex-related differences. Hormonal influences, particularly the immunomodulatory effects of estrogens and androgens, can shape immune responses and may modulate vascular function. Moreover, genetic and epigenetic variations, including differences in hsa-miRs regulation, could further contribute to divergent inflammatory and cardiovascular pathways between women and men.

Finally, we identified several biological factors influencing hsa-miR expression over time. Among them, age, disease duration, sex and several related cardiometabolic conditions such as hypertension or BMI had a significant effect. These findings reinforce that hsa-miR expression is modulated by specific conditions and highlight the unexplored significance of disease duration in hsa-miR regulation and disease pathogenesis (47–49).

Despite rigorous statistical analyses, including lineal mixed-models and machine learning approaches that accounted for known confounders and were cross-validated, several limitations should be acknowledged. First, although the sample size is substantial for a longitudinal RA cohort, it may still have limited the detection of certain associations, particularly regarding mortality. Therefore, validation in independent and larger cohorts is essential to confirm the robustness and clinical relevance of these findings. Second, the generalizability of the results may be limited, as the cohort consisted exclusively of European Caucasian patients. Third, stratification of the ASCVD progression endpoint inevitably reduced the sample size and may have limited statistical power. Nevertheless, even with the relatively small numbers of carotid plaque progression and incident ASCVD events individually, several hsa-miRs remained significantly associated with these outcomes. This consistency reinforces their potential role in both subclinical and clinical stages of atherosclerotic progression. Also, while stratifying all analyses by patients’ inflammatory status (low vs. high disease activity) would have been of interest, the relatively small subgroup sizes would have compromised the robustness and reliability of the results. To mitigate this limitation, all models were adjusted for DAS28 (specifically DAS28-ESR), thereby accounting for disease activity and minimizing potential bias related to inflammation. Finally, although hsa-miR quantification is widely used and accepted, the inherent measurement variability may limit the extrapolation of these results to other settings using different quantification techniques. Nonetheless, the use of a standardized internal control, such as hsa-miR-16, strengthens the reliability and validity of our findings.

## Conclusions

This study demonstrates that a substantial proportion of patients with RA experience ASCVD progression and elevated mortality from cardiovascular events or infections, despite advances in disease control likely due to the widespread use of biological therapies. Our findings identify a panel of hsa-miRs as a promising biomarker for predicting ASCVD progression and mortality in RA. With further validation, this hsa-miR panel could be integrated into clinical practice, enabling refined risk stratification and personalized therapeutic strategies to mitigate ASCVD-related complications and improve long-term outcomes in RA.

## Data Availability

The raw data supporting the conclusions of this article will be made available by the authors, without undue reservation.
