# Supplementary material for: Atherosclerotic cardiovascular disease and mortality in a cohort of patients with rheumatoid arthritis: a prospective study investigating microRNAs as predictors of atherosclerosis and mortality
Source: Front Immunol. 2025 Oct 8;16:1667553. doi: 10.3389/fimmu.2025.1667553 (PMC12540091; doi:10.3389/fimmu.2025.1667553)
Supplement: Supplementary file 1 [file Table1.docx]

| **Basal** | | | | |
| --- | --- | --- | --- | --- |
|  | **Overall cohort**  **(n = 235)** | **Men patients**  **(n = 85)** | **Women patients**  **(n = 150)** | **p** |
| **Clinical and demographic** | | | |  |
| Age  (years, IQR) | 57 (49 – 67) | 57 (50 – 67) | 57 (48 – 67) | 0.74 |
| Sex, female  (n, %) | 150, 64% |  |  |  |
| Body mass index  (kg/m2, IQR) | 27.1 (23.6 – 30.8) | 28.3 (25.8 – 30.6) | 26.6 (23.0 – 31.7) | 0.11 |
| Hypertension  (n, %) | 138, 59% | 61, 72% | 77, 51% | <0.001 |
| T2DM  (n, %) | 27, 11% | 10, 12% | 17, 11% | 1 |
| Dyslipidaemia  (n, %) | 96, 41% | 37, 44% | 59, 39% | 0.62 |
| Current smoker  (n, %) | 61, 26% | 21, 25% | 40, 27% | 0.86 |
| **Lipidic profile** | | | |  |
| Total cholesterol  (mg/dL, IQR) | 203 (182.5 – 227.5) | 191 (173 – 219) | 206 (185 – 233) | 0.01 |
| LDL-C  (mg/dL, IQR) | 114 (99 – 135.5) | 116 (101 – 136) | 114 (96 – 135) | 0.45 |
| HDL-C  (mg/dL, IQR) | 65 (52 – 76) | 52 (43 – 65) | 69 (61 – 82) | <0.001 |
| TG  (mg/dL, IQR) | 94 (70 – 127.5) | 96 (74 – 130) | 87 (65 – 125) | 0.14 |
| **RA disease features and treatments** | | | |  |
| Disease duration  (years, IQR) | 6 (2 – 13) | 6 (2 – 11) | 7.5 (3 – 13) | 0.09 |
| DAS28-ESR  (median, IQR) | 3.42 (2.6 – 4.4) | 3 (2.4 – 3.6) | 3.6 (2.8 – 4.7) | <0.001 |
| - Remission (n, %) | 59, 25% | 31, 36% | 28, 19% | 0.004 |
| - Low activity (n, %) | 45, 19% | 22, 26% | 23, 15% | 0.07 |
| - Moderate activity (n, %) | 106, 45% | 28, 33% | 78, 52% | 0.007 |
| - High activity (n, %) | 25, 11% | 4, 5% | 21, 14% | 0.04 |
| DAS28-CRP  (median, IQR) | 2.1 (1.3 – 3.0) | 1.4 (1.1 – 2.4) | 2.3 (1.5 – 3.2) | <0.001 |
| RF+  (%, n) | 173, 74% | 64, 75% | 109, 73% | 0.78 |
| ACPA+  (%, n) | 167, 71% | 58, 68% | 109, 73% | 0.57 |
| ESR  (mm/h, IQR) | 30 (18 – 50) | 27 (16 – 45) | 32 (19 – 54) | 0.04 |
| CRP  (mg/dL, IQR) | 0.40 (0.2 – 0.9) | 0.5 (0.2 – 0.9) | 0.4 (0.2 – 0.9) | 0.56 |
| csDMARDs  (n, %) | 175, 74% | 67, 79% | 108, 72% | 0.32 |
| Biological agents  (n, %) | 46, 20% | 12, 14% | 34, 23% | 0.16 |
| - JAK inhibitors (n, %) | 0, 0% | 0, 0% | 0, 0% | 1 |
| NSAIDs  (n, %) | 137, 58% | 50, 59% | 87, 58% | 1 |
| Corticoids  (n, %) | 122, 52% | 42, 49% | 80, 53% | 0.66 |
| Lipid-lowering therapies  (n, %) | 42, 18% | 17, 20% | 25, 17% | 0.64 |
| **Atherosclerosis and CV disease variables** | | | |  |
| Plaque presence  (n ,%) | 92, 39% | 45, 53% | 47, 31% | 0.002 |
| CV event  (n, %) | 18, 8% | 15, 18% | 3, 2% | <0.001 |

**Supplementary Table 1:** General characteristics of the overall cohort and men and women with RA at 5 years.

IQR = interquartile range, T2DM = type 2 diabetes mellitus, LDL-C = low-density lipoprotein cholesterol, HDL-C = high-density lipoprotein cholesterol, TG = triglycerides, DAS28 = disease activity score, RF = rheumatoid factor, ACPA = anti-citrullinated peptide antibodies, ESR = erythrocyte sedimentation rate, CRP = C-reactive protein, csDMARDS = conventional synthetic disease modifying antirheumatic drugs, NSAIDs = non-steroidal anti-inflammatory drugs, JAK = Janus Kinase, CV= cardiovascular

| **Follow-up** | | | | |
| --- | --- | --- | --- | --- |
|  | **Overall cohort**  **(n = 148)** | **Men patients**  **(n = 47)** | **Women patients**  **(n = 101)** | **p** |
| **Clinical and demographic** | | | |  |
| Age  (years, IQR) | 63 (56 – 69) | 61.50 (56 – 67) | 63 (55 – 70) | 0.44 |
| Sex, female  (n, %) | 101, 68.24% |  |  |  |
| Body mass index  (kg/m2, IQR) | 26.53 (23.25 – 30.76) | 27.43 (25.53 – 31.01) | 26.12 (22.75 – 30.58) | 0.06 |
| Hypertension  (n, %) | 95, 64.20% | 31, 66% | 64, 63.33% | 0.06 |
| T2DM  (n, %) | 23, 16% | 10, 21.27% | 13, 13% | 0.35 |
| Dyslipidaemia  (n, %) | 83, 56.10% | 28, 60% | 55, 54% | 0.63 |
| Current smoker  (n, %) | 28, 19% | 9, 19% | 19, 19% | 1 |
| **Lipidic profile** | | | |  |
| Total cholesterol  (mg/dL, IQR) | 199.5 (170.8 – 228) | 183 (160.2 – 213.8) | 203 (182.2 – 231.2) | 0.01 |
| LDL-C  (mg/dL, IQR) | 110 (90 – 132) | 103.50 (92.25 – 130) | 113 (90 – 135) | 0.53 |
| HDL-C  (mg/dL, IQR) | 61.50 (51 – 78) | 53.50 (41.75 – 66) | 68 (56.25 – 82.75) | <0.001 |
| TG  (mg/dL, IQR) | 98 (77 – 140) | 100.50 (77.75 – 133) | 95.5 (75.5 – 148.5) | 0.86 |
| **RA disease features and treatments** | | | |  |
| Disease duration  (years, IQR) | 14 (10 – 20) | 14 (9 – 18.75) | 14.50 (10 – 21) | 0.18 |
| DAS28-ESR  (median, IQR) | 2.74 (2.23 – 3.68) | 2.67 (2.11 – 3.65) | 2.75 (2.30 – 3.67) | 0.30 |
| - Remission (n, %) | 58, 39% | 20, 43% | 38, 38% | 0.33 |
| - Low activity (n, %) | 40, 27% | 13, 28% | 27, 27% | 0.01 |
| - Moderate activity (n, %) | 48, 32% | 12, 26% | 36, 36% | 0.22 |
| - High activity (n, %) | 2, 1% | 1, 2% | 1, 1% | 0.31 |
| DAS28-CRP  (median, IQR) | 1.95 (1.50 – 2.66) | 1.87 (1.54 – 2.31) | 2.00 (1.50 – 2.85) | 0.66 |
| RF+  (%, n) | 118, 80% | 36, 77% | 82, 81% | 0.15 |
| ACPA+  (%, n) | 116, 78% | 37, 79% | 79, 78% | 1 |
| ESR  (mm/h, IQR) | 24.50 (13.75 – 42) | 24 (14.25 – 40) | 26 (12.25 – 42.75) | 0.73 |
| CRP  (mg/dL, IQR) | 0.20 (0.10 – 0.625) | 0.30 (0.10 – 0.70) | 0.20 (0.10 – 0.58) | 0.25 |
| csDMARDs  (n, %) | 84, 57% | 23, 49% | 61, 60% | 0.92 |
| Biological agents  (n, %) | 71, 48% | 20, 43% | 51, 50.5% | 0.92 |
| - JAK inhibitors (n, %) | 11, 7% | 6, 13% | 5, 5% | 0.16 |
| NSAIDs  (n, %) | 18, 12% | 6, 13% | 12, 12% | 0.71 |
| Corticoids  (n, %) | 45, 30% | 9, 19% | 36, 36% | 1 |
| Lipid-lowering therapies  (n, %) | 39, 26% | 13, 28% | 26, 26% | 0.21 |
| **Atherosclerosis and CV disease variables** | | | |  |
| Plaque presence  (n ,%) | 73, 49% | 30, 64% | 43, 43% | 0.01 |
| CV event  (n, %) | 20, 14% | 10, 21% | 10, 10% | 0.03 |

**Supplementary Table 2:** General characteristics of the overall cohort and men and women with RA at 5 years.

IQR = interquartile range, T2DM = type 2 diabetes mellitus, LDL-C = low-density lipoprotein cholesterol, HDL-C = high-density lipoprotein cholesterol, TG = triglycerides, DAS28 = disease activity score, RF = rheumatoid factor, ACPA = anti-citrullinated peptide antibodies, ESR = erythrocyte sedimentation rate, CRP = C-reactive protein, csDMARDS = conventional synthetic disease modifying antirheumatic drugs, NSAIDs = non-steroidal anti-inflammatory drugs, JAK = Janus Kinase, CV= cardiovascular

|  | **Basal characteristics of RA patients who were alive at follow-up (n = 148)** | **Basal characteristics of RA patients who deceased during follow-up (n = 35)** | **p** |
| --- | --- | --- | --- |
| **Clinical and demographic** | | | |
| Age  (years, IQR) | 55 (46.8 – 62) | 73 (64 – 77) | <0.001 |
| Sex, female  (n, %) | 101, 68.24% | 16, 46% | 0.02 |
| Body mass index  (kg/m2, IQR) | 26.6 (23.5 – 30.1) | 30 (27.5 – 32.2) | 0.003 |
| Hypertension  (n, %) | 76, 51.35% | 29, 83% | 0.001 |
| T2DM  (n, %) | 10, 6.75% | 8, 23% | 0.01 |
| Dyslipidaemia  (n, %) | 51, 34.45% | 18, 51% | 0.10 |
| Current smoker  (n, %) | 43, 29% | 5, 14% | 0.12 |
| **Lipidic profile** | | | |
| Total cholesterol  (mg/dL, IQR) | 202.5 (183.8 – 225.2) | 195 (183 – 226) | 0.81 |
| LDL-C  (mg/dL, IQR) | 115 (99 – 134.2) | 114 (99.5 – 132) | 0.75 |
| HDL-C  (mg/dL, IQR) | 66 (56 – 77) | 65 (48 – 73) | 0.33 |
| TG  (mg/dL, IQR) | 87.50 (65 – 119) | 101 (76 – 143) | 0.06 |
| **RA disease features and treatments** | | | |
| Disease duration  (years, IQR) | 6 (3 – 13) | 10 (4 – 15.5) | 0.27 |
| DAS28-ESR  (median, IQR) | 3.45 (2.58 – 4.29) | 3.2 (2.8 – 3.8) | 0.67 |
| - Remission (n, %) | 37, 25% | 7, 20% | 0.69 |
| - Low activity (n, %) | 27, 18% | 11, 31% | 0.13 |
| - Moderate activity (n, %) | 68, 46% | 15, 43% | 0.89 |
| - High activity (n, %) | 16, 11% | 2, 6% | 0.55 |
| DAS28-CRP  (median, IQR) | 2.13 (1.30 – 3.06) | 1.7 (1.2 – 2.2) | 0.03 |
| RF+  (%, n) | 110, 74.3% | 29, 83% | 0.40 |
| ACPA+  (%, n) | 109, 74% | 27, 77% | 0.83 |
| ESR  (mm/h, IQR) | 27.50 (15 – 45.25) | 37 (29.5 – 61) | 0.004 |
| CRP  (mg/dL, IQR) | 0.30 (0.20 – 0.80) | 0.7 (0.3 – 1.1) | 0.01 |
| csDMARDs  (n, %) | 111, 75% | 29, 83% | 0.44 |
| Biological agents  (n, %) | 37, 25% | 4, 11% | 0.13 |
| - JAK inhibitors | 0, 0% | 0, 0% | 1 |
| NSAIDs  (n, %) | 93, 63% | 17, 49% | 0.17 |
| Corticoids  (n, %) | 70, 47.3% | 22, 63% | 0.14 |
| Lipid-lowering therapies  (n, %) | 22, 15% | 9, 26% | 0.20 |
| **Atherosclerotic cardiovascular disease progression** | | | |
| Carotid plaque presence  (n, %) | 44, 30% | 26, 74% | <0.001 |
| CV events  (n, %) | 8, 5.4% | 7, 20% | 0.01 |

**Supplementary Table 3:** General characteristics of the patients who remained alive and those who died after the follow-up period.

IQR = interquartile range, T2DM = type 2 diabetes mellitus, LDL-C = low-density lipoprotein cholesterol, HDL-C = high-density lipoprotein cholesterol, TG = triglycerides, DAS28 = disease activity score, RF = rheumatoid factor, ACPA = anti-citrullinated peptide antibodies, ESR = erythrocyte sedimentation rate, CRP = C-reactive protein, csDMARDS = conventional synthetic disease modifying antirheumatic drugs, NSAIDs = non-steroidal anti-inflammatory drugs, JAK = Janus Kinase, CV= cardiovascular

| **hsa-miRs as predictors of carotid plaque presence progression** | | |
| --- | --- | --- |
| ***Women*** | | |
|  | **OR (95% CI), p-value** | **AIC** |
| Initial model |  | 107.41 |
| hsa-miR-24 | 0.52 (0.28 – 0.88), p=0.02 | 103.35 |
| hsa-miR-146 | 0.61 (0.38 – 0.94), p=0.03 | 104.45 |
| hsa-miR-Let7a | 0.56 (0.32 – 0.90), p=0.03 | 103.65 |
| hsa-miR-425 | 0.51 (0.25 – 0.98), p=0.05 | 105.36 |

**Supplementary Table 4:** Logistic regression models evaluating the association of baseline expression levels of the selected miRs with the progression of carotid plaque presence. Models were adjusted for age, BMI, disease duration, hypertension status, T2DM, dyslipidaemia, DAS28, RA treatment and lipid-lowering therapies.

BMI = body mass index, csDMARDs = conventional synthetic disease modifying antirheumatic drugs, T2DM = type 2 diabetes mellitus, AT-CVD progression = atherosclerosis and cardiovascular disease progression.

| **hsa-miRs as predictors of new cardiovascular events** | | |
| --- | --- | --- |
| ***Overall*** | | |
|  | **OR (95% CI), p-value** | **AIC** |
| Initial model |  | 82.02 |
| hsa-miR-146 | 0.52 (0.24 – 0.98), p=0.05 | 78.86 |
| hsa-miR-Let7a | 0.52 (0.23 – 0.97), p=0.05 | 79.81 |
| has-miR-155-5p | 0.50 (0.24 – 0.92), p=0.04 | 78.78 |

**Supplementary Table 5:** Logistic regression models evaluating the association of baseline expression levels of the selected miRs with incident cardiovascular events. Models were adjusted for age, sex, BMI, disease duration, hypertension status, T2DM, dyslipidaemia, DAS28, RA treatment and lipid-lowering therapies.

BMI = body mass index, csDMARDs = conventional synthetic disease modifying antirheumatic drugs, T2DM = type 2 diabetes mellitus, AT-CVD progression = atherosclerosis and cardiovascular disease progression.

| **Full Model** | **OR** | **p-value** |
| --- | --- | --- |
| Age | 2.56 | 0.004 |
| Sex | 3.42 | 0.014 |
| BMI | 0.60 | 0.052 |
| Disease duration | 1.52 | 0.109 |
| Hypertension | 1.41 | 0.586 |
| Dyslipidemia | 1.66 | 0.342 |
| T2DM | 2.19 | 0.176 |
| csDMARDs | 0.54 | 0.215 |
| NSAIDs | 0.047 | < 0.001 |
| Corticoids | 1.20 | 0.67 |
| Biologic therapies | 2.16 | 0.14 |
| Lipid-lowering therapies | 2.57 | 0.096 |
| **Initial Model** | **OR** | **p-value** |
| Age | 3.13 | < 0.001 |
| Sex | 2.82 | 0.025 |
| BMI | 0.65 | 0.078 |
| NSAIDs | 0.05 | < 0.001 |
| T2DM | 2.88 | 0.01 |
| Biologic therapies | 3.03 | 0.007 |
| Lipid-lowering therapies | 3.72 | 0.059 |

**Supplementary Table 6:** Generalized linear-effect models adjusted to select the most influential confounders. The dependent variable was AT–CVD progression.

BMI = body mass index, csDMARDs = conventional synthetic disease modifying antirheumatic drugs, T2DM = type 2 diabetes mellitus, AT-CVD progression = atherosclerosis and cardiovascular disease progression.

|  | **β** | **p-value** |
| --- | --- | --- |
| **Dependent Variable: hsa-miR-24** | | |
| Age | -0.19 | 0.01 |
| Sex | 0.07 | 0.59 |
| BMI | 0.08 | 0.19 |
| Disease duration | -0.02 | 0.81 |
| Dyslipidemia | -0.32 | 0.01 |
| Hypertension | 0.04 | 0.76 |
| T2DM | -0.06 | 0.77 |
| **Dependent Variable: hsa-miR-146** | | |
| Age | -0.18 | 0.001 |
| Sex | 0.04 | 0.78 |
| BMI | 0.08 | 0.21 |
| Disease duration | -0.06 | 0.37 |
| Dyslipidemia | -0.31 | 0.01 |
| Hypertension | 0.09 | 0.49 |
| T2DM | -0.17 | 0.39 |
| **Dependent Variable: hsa-miR-Let7a** | | |
| Age | -0.12 | 0.13 |
| Sex | 0.08 | 0.55 |
| BMI | 0.07 | 0.26 |
| Disease duration | -0.15 | 0.04 |
| Dyslipidemia | -0.25 | 0.06 |
| Hypertension | 0.14 | 0.035 |
| T2DM | -0.19 | 0.36 |
| **Dependent Variable: hsa-miR-425** | | |
| Age | -0.13 | 0.09 |
| Sex | 0.05 | 0.72 |
| BMI | 0.17 | 0.008 |
| Disease duration | -0.15 | 0.04 |
| Dyslipidemia | -0.21 | 0.12 |
| Hypertension | -0.01 | 0.93 |
| T2DM | -0.05 | 0.79 |
| **Dependent Variable: hsa-miR-451** | | |
| Age | 0.07 | 0.33 |
| Sex | -0.26 | 0.04 |
| BMI | 0.12 | 0.06 |
| Disease duration | -0.08 | 0.26 |
| Dyslipidemia | 0.17 | 0.18 |
| Hypertension | 0.06 | 0.66 |
| T2DM | 0.14 | 0.49 |
| **Dependent Variable: hsa-miR-155** | | |
| Age | -0.12 | 0.14 |
| Sex | -0.03 | 0.84 |
| BMI | 0.003 | 0.96 |
| Disease duration | -0.16 | 0.04 |
| Dyslipidemia | -0.24 | 0.08 |
| Hypertension | 0.06 | 0.66 |
| T2DM | -0.12 | 0.56 |

**Supplementary Table 7**: Linear mixed-effects models for determining which biological variables influenced changes in has-miR expression.

BMI = body mass index, T2DM = type 2 diabetes mellitus


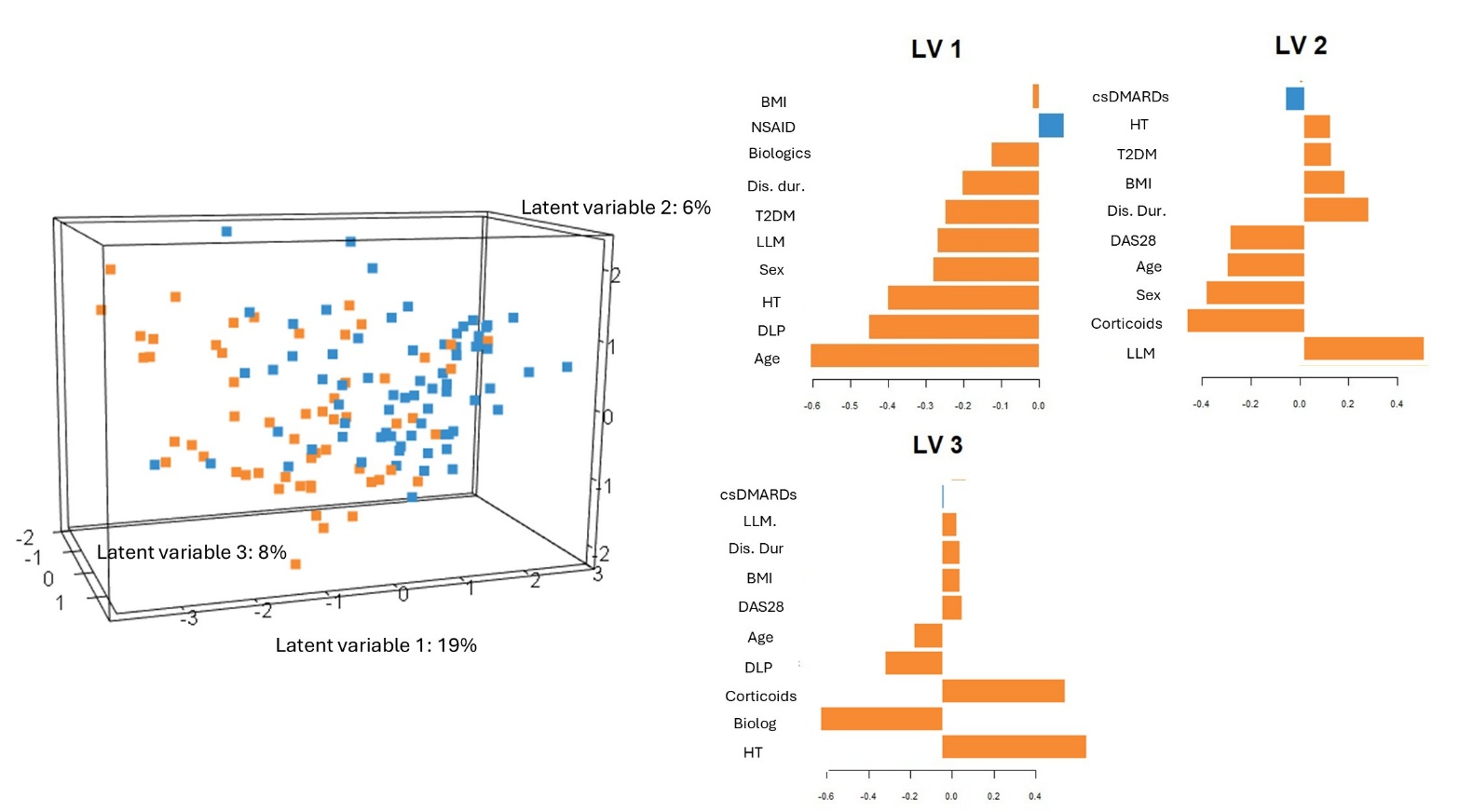


**Supplementary Figure 1:** Graphical representation of the sPLS-DA model for discerning between those who have experienced the cardiovascular endpoint and those who not, including all confounders (without hss-miRs), along with the feature importance of each LV.

sPLS-DA = sparse partial least square discriminant analysis, LV = Latent variable


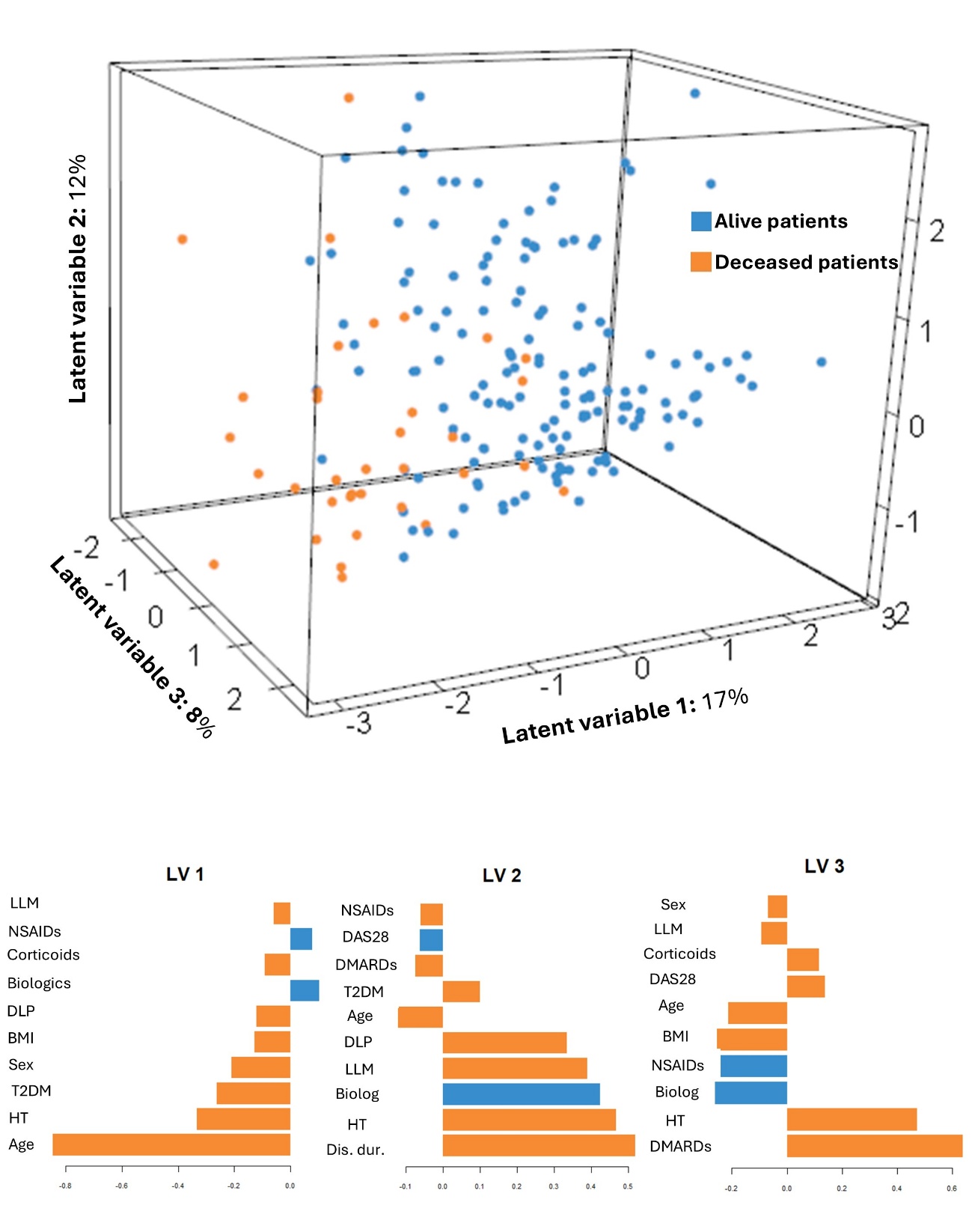


**Supplementary Figure 2:** Graphical representation of the sPLS-DA model to discern between alive and dead patients including all confounders (without hsa-miRs), along with the feature importance of each LV.

sPLS-DA = sparse partial least square discriminant analysis, LV = Latent variable
